# A quality control circle process to improve enteral nutrition feeding support in discharged patients with colorectal cancer

**DOI:** 10.3389/fnut.2023.1191804

**Published:** 2023-07-19

**Authors:** Youyan Lin, Xinyan Yu, Xiaoyu Ni, Wenxi Shu, Qiuhong Zheng, Fengzhou Chen, Bo Zhang, Chao Xu, Ling Liu, Yi Lu

**Affiliations:** ^1^Department of Integrated Chinese and Western Medicine, Zhejiang Cancer Hospital, Hangzhou Institute of Medicine (HIM), Chinese Academy of Sciences, Hangzhou, Zhejiang, China; ^2^Integrated Traditional Chinese and Western Medicine Oncology Laboratory, Key Laboratory of Traditional Chinese Medicine of Zhejiang Province, Hangzhou, Zhejiang, China; ^3^Second Clinical College of Zhejiang Chinese Medical University, Hangzhou, China; ^4^Department of Clinical Nutrition, Zhejiang Cancer Hospital, Hangzhou Institute of Medicine (HIM), Chinese Academy of Sciences, Hangzhou, Zhejiang, China

**Keywords:** quality control circle, enteral nutrition, nutritional support, home nutrition therapy, colorectal cancer

## Abstract

Correct usage and maintenance of the enteral nutrition feeding pump system is always a challenge in nutrition support for patients with colorectal cancer (CRC). However, there are few studies on the sustained accuracy improvement of the enteral nutrition feeding system in discharged CRC patients. Here, we established a seven-month quality control circle (QCC) activity with the theme of improving the performance of home enteral feeding pumps (EFP) and examined the effect of QCC activity on the nutritional state and quality of life in discharged CRC patients. We enrolled 100 discharged CRC patients treated with home enteral nutrition from Zhejiang Cancer Hospital between March 2020 and December 2021. The patients were randomly split into two research groups: one participated in the QCC activity (*n* = 50) and the other did not (*n* = 50). QCC analysis indicated that the top 3 causes of EFP inaccurate usage are the simple and boring contents of training, various types of pumps, no examination rules, and lack of management. Furthermore, both intra- and inter-group comparisons showed that QCC significantly improved the patients’ pass rate of nutrition pump operation from 52 to 70% after 1-month of activity, which gradually improved and got the highest (90%) after 3 months (*p* < 0.05). Interestingly, the established QCC activity significantly increased the patient-generated subjective global assessment (PG-SGA) and Barthel index (BI) scores, body fat mass (BFM) and superior longitudinal muscle (SLM) by intra- and inter-group comparisons. In this study, we clarified the main causes of inaccurate EFP usage and established a QCC process to improve the pass rate of EFP usage. It finally leads to the improvement of nutritional state and quality of life in CRC patients.

## Introduction

Malnutrition is common in patients with colorectal cancer (CRC) due to factors such as the local effect of the tumor on intestinal function, body metabolism caused by the tumor, and antitumor therapies (1). Since 1946, the important role of medical nutrition therapy has been highlighted in patients with CRC, and now it becomes a topic of much relevance in cancer research (2, 3). Home nutrition support is gradually emerging as the development of nutritional support equipment, the reduction in the number of hospital beds, the rise in healthcare costs, and especially the increasing number of discharged patients who need long-term nutrition support. Home nutrition support includes home enteral nutrition (HEN) and home parenteral nutrition (HPN) (4). HPN requires precise technical specifications, has high medical costs, and is prone to complications, some studies even showed that 30 days of home parenteral nutrition support after hospital discharge increases readmission rates (5). Home parenteral nutrition has been somewhat limited in its area of use. ESPEN guideline advocates enteral nutrition (EN) as long as the patient’s intestinal function allows with the goal of improving body weight, functional status or quality of life (QoL) (6, 7). Routes of home enteral nutrition include oral, nasal tubes, and percutaneous catheter feeding. For continuous feeding and high-energy feeding, it has been recommended medical nutrition therapy using pumps for discharged patients (8). The enteral feeding pump (EFP) is a microcomputer (MCU)-controlled input device that can precisely control the infusion rate of enteral nutrition solution, maintain the relative sterility of the nutrition solution, the stability of food osmolality, and the constant temperature and rate (9). EFP has been confirmed to reduce complications during nasal feeding and improve patient comfort and satisfaction (9). However, feeding pump inaccuracy commonly occurs when discharged CRC patients handle it at home. A volume per day provided by enteral nutrition significantly affects the efficacy of EN delivery including energy delivery, the proportion of calorie delivery, completion of 80% energy delivery and protein delivery. Accuracy is critically important since small variances in volume can result in a significant change in nutrition delivery (10, 11). Appropriate enteral nutrition brought beneficial effects on the quality of life of cancer patients, while inappropriate enteral nutrition led to an inverse outcome and generated detrimental impacts (12). Therefore, it is important to manage and improve the accurate usage of EFP, especially for discharged CRC patients who lack instant guidance and supervision from doctors.

The Quality Control Circle (QCC) has outgrown its original scope in business management. It is a problem-solving method that provides means to better achieve goals and improvements in clinical practice. It is made up of clinicians who agree to meet periodically to identify, analyze, and solve problems in order to increase quality, reduce management costs and increase the efficiency of medical services (13). The majority of QCC activities in medical studies focused on the themes of safety (29.5%) and patient care (22.7%) (14). The top three themes of QCC activities in medical care are reducing the incidence of accidental falls, pressure ulcers, and decreasing pharmacy dispensing errors (15). Recently, some studies focused on the effects of QCC activities on cancer patients, including the improvement of drug intervention effectiveness (16) and immune function (17), and cancer pain control (18, 19). However, few studies explored the role of QCC activities in the improvement of nutritional status in cancer patients (17), and no study has yet explored the effect of QCC activities on EFP accuracy in cancer patients.

Extensive research indicated a protective role of nutrition in the development of colon cancer (20). Recently, a preliminary report on 1,000 outpatients claimed that the incidence of nutritional risk in oncology patients is 33.8%, and malnutrition incidence is 39.7%, which were assessed by the Nutrition Risk Screening 2002 questionnaire (21), which is closely related to cancer cachexia and directly affects the clinical outcome (22). Appropriate and moderate medical nutrition therapy benefited colorectal cancer patients, including reducing postoperative complications, shorting hospital stay, decreasing the cost of hospitalization and improving postoperative outcomes (23–26). Here, we established a QCC process and conduct a series of activities to improve EFP accuracy at home for discharged CRC patients. Since March 2020, 100 CRC patients with EFP feeding in our hospital were randomly split into two groups: the experimental group including those who participated in the QCC activity (*n* = 50), and the control group including those who did not (*n* = 50). QCC analysis showed that the top 3 causes of EFP inaccurate usage are the simple and boring contents of training, various types of pumps, no examination rules, and lack of management. Also, a before-and-after comparative study was designed to evaluate the effect of the QCC on EFP accuracy and nutrition state of discharged patients with CRC. We evaluated patients’ pass rate of nutrition pump operation, nutritional status indicators, patient-generated subjective global assessment (PG-SGA), and Barthel Index (BI) for Activities of Daily Living (ADL) in colorectal cancer patients before and after the application of the QCC.

## Materials and methods

### Patients

This study was approved by the Institutional Review Board of the Cancer Hospital of the University of Chinese Academy of Sciences (Zhejiang Cancer Hospital) and written informed consent were obtained from all patients. This study complied with the tenets of the Declaration of Helsinki and all methods were performed by relevant protocol. 100 discharged CRC patients treated with home enteral nutrition by EFP feeding were enrolled from Zhejiang Cancer Hospital between March 2021 and December 2022. The inclusion criteria were: (1) CRC discharged patients received nutrition support through EFP feeding, (2) Patients were conscious, had no organ failure, life expectancy >1 year, (3) The patients and their families had voluntarily participated in the QCC activity and the follow-up study. We set the exclusion criteria as follows: (1) Those who participated in other clinical trials, (2) Those with auditory neuropathy, central nervous system diseases, or psychiatric diseases; (3) Those who stopped the usage of EFP feeding because of outside reasons (non-study reasons), (4) Those who were lost to follow-up. In total, 43 women and 57 men between 60 and 85 years old were evaluated in this study. The patients were randomly divided into a control group and an experimental group (50 cases for each group), with no patient withdrawal during the activity. There was no statistically significant difference in sex, age, and hospitalization days between the two groups (*p* > 0.05). More details about the characteristics of the patients are shown in Table 1.

**Table 1:** Patient characteristics.

Characteristic	Total (*n* = 100)	Control group (*n* = 50)	Observation group (*n* = 50)	*p* value
Age, Mean (IQR)	68.28 (60–85)	67.56 (60–83)	69.12 (60–85)	0.817
Gender, *n* (%)				
Male	57 (57%)	29 (58%)	28 (56%)	0.633
Female	43 (43%)	21 (42%)	22 (44%)	
Marital status, *n* (%)				
Married	89 (89%)	45 (90%)	44 (88%)	0.137
Single (including divorced and widowed)	11 (11%)	5 (10%)	6 (12%)	
Education, *n* (%)				
≤Primary school	42 (42%)	22 (44%)	20 (40%)	0.561
Middle school and high school	45 (45%)	21 (42%)	24 (48%)	
≥Bachelor	13 (13%)	7 (14%)	6 (12%)	
Hospitalization days (IQR)	6 (1–11)	5.5 (1–10)	6.4 (1–11)	0.354
Payment, *n* (%)				
Insurance	99 (99%)	50 (100%)	49 (98%)	0.762
Self-pay	1 (1%)	0	1 (2%)	

IQR, interquartile range.

### Study design

The QCC program was carried out according to the QCC theory (13), and the basic steps and flow of the QCC program could be found in Supplementary Figure S1. Briefly, there were three stages in the study (27). Firstly, we established the QCC team and selected the operation accuracy improvement of enteral nutrition feeding in discharged patients with colorectal cancer as our goal. Secondly, the current status of home EFP operation and related reasons causing low accuracy were analyzed, we build several strategies and implemented them. Thirdly, the results were verified and the protocol was standardized. During this process, PDCA (plan, do, check and act) circulation was applied for monthly activities and revisions until the results were verified.

### QCC activity

#### QCC team

The QCC program was composed of nine members aged 28–56 years old, with an average age of 34 years old. The QCC team consisted of one dietitian, 2 senior oncologists (major in the gastrointestinal tumor), 4 nurses (including 1 head nurse) of some clinical departments, and 2 EFP operation professionals. They acted as project director, counselor, circle head and circle members, respectively.

#### Theme selection

The circle members collected problems that appeared in their work or clinical practice, and extracted several themes from the opinions in a systematic way. Then every QCC member voted to choose the main target using the evaluation method. That is, all members rated the theme items, and then the scores of the themes are summed and averaged, and the ones with the highest score were the activity themes of the current quality control circle. In this process, four dimensions were concerned: leaders’ concern, importance, urgency, and circle capability. The weight of the above four dimensions were 36, 29, 16, and 19%, respectively based on the scores rated by QCC members using the L-type matrix (16). According to the different extent, three grades for each dimension were classified with a score of 5, 3, and 1 corresponding to each grade. The higher score indicated a higher extent. Leaders’ concern was classified into “deeply concerned,” “concerned,” and “not concerned,” importance was classified into “very important,” “important” and “not important,” urgency was classified as “solve it as soon as possible,” “solve it next time” and “solve it after 6 months,” circle capability was classified into “can solve it by ourselves,” “need to cooperate with another department” and “need to cooperate with multiple departments.” Finally, the theme of “Improvement of home EFP operation accuracy in discharged patients with colorectal cancer” was adopted. The evaluation for this theme was based on “nutrition pump operation pass rate” [total operation pass rate = (total number of qualified items ÷ total number of all items) × 100%].

#### Process and standardization

The whole QCC activity lasted for 7 months. It was held once a week and 2 h each time. The assignment for the seven-month QCC activity was as follows: at the 1st month, we set up the quality control circle curriculum, elected a circle leader, picked a circle name, and fixed a theme. In the 2nd–3rd month, we fixed the activity plan, grasped the current situation, set goals, and proposed countermeasures. In the 4th–7th month, implementation was carried out.

**Figure 1:**
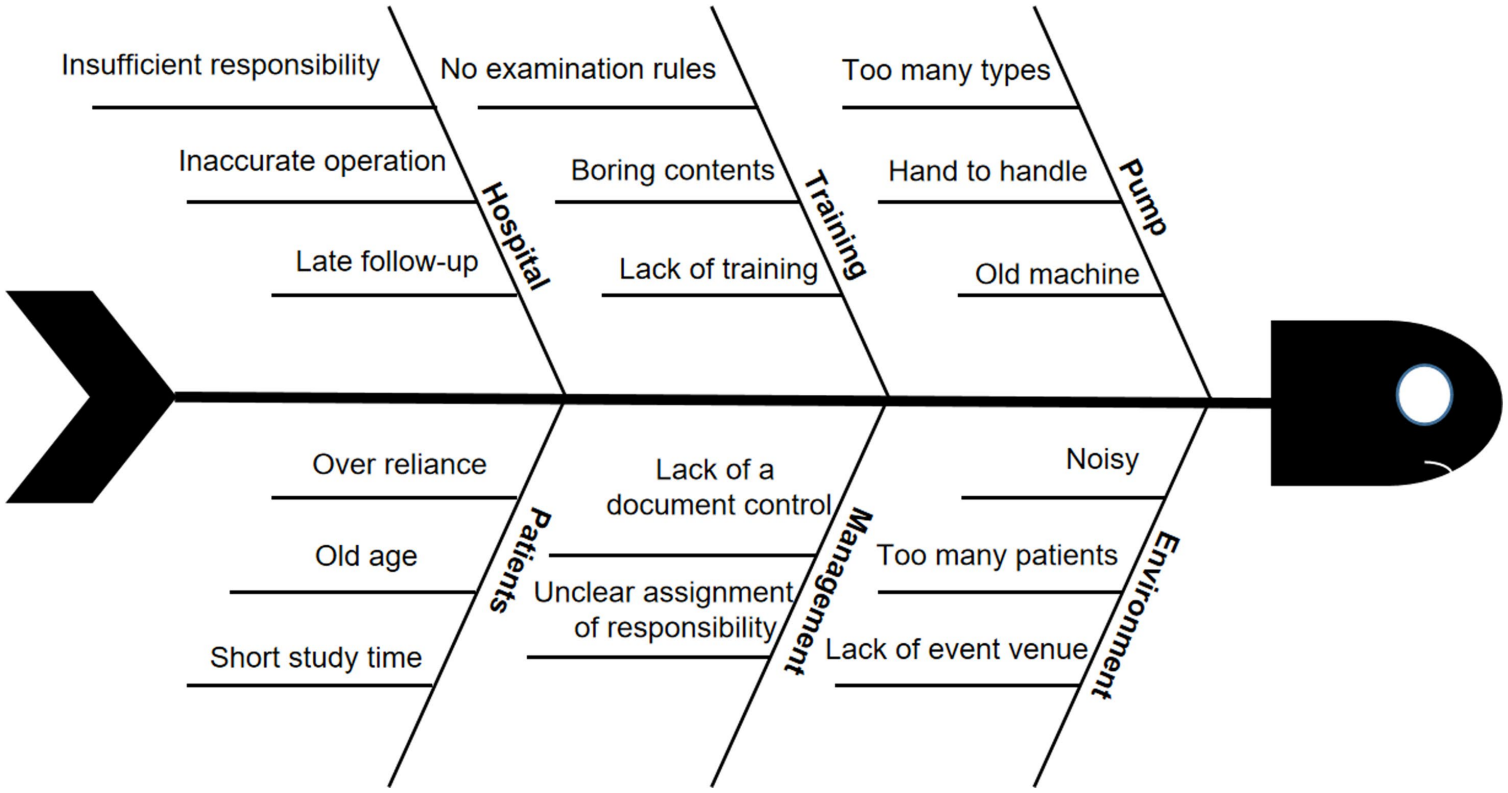
The reasons for the low feeding accuracy of enteral feeding pump (EFP) demonstrated in a fishbone diagram.

**Figure 2:**
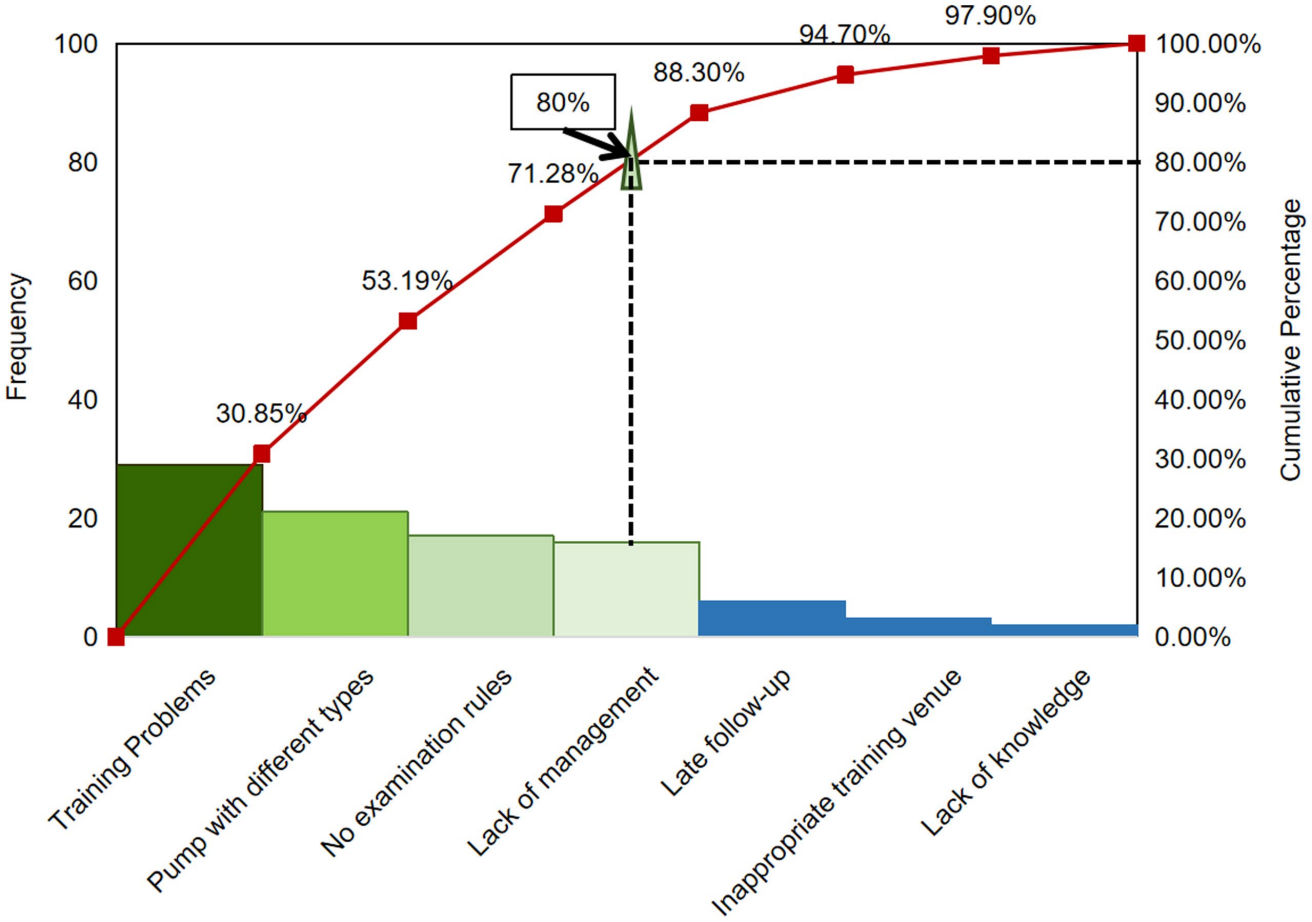
Pareto analysis of the reasons for low feeding accuracy of enteral feeding pump (EFP) discharged patients with colorectal cancer before QCC activity (*n* = 100).

The QCC program started with the understanding of the status of the operation of home EFP by discharged patients with colorectal cancer before QCC activity. By investigating the current situation of 94 patients, the possible reasons for the low accuracy of home EFP operation were demonstrated in the fishbone diagram (Figure 1). There were five main reasons were voted out by nine QCC members: alarm handling, catheter routine maintenance, infusion equipment, knowledge overview, nutrition monitoring. According to the survey results, the total operation pass rate was only 58.2% before the QCC activity, and the cumulative error rate of each item was 45.7, 79.8, 90.3, and 97.7%, respectively. Next, we set the goal of the QCC activity and take the improvement of the pass rate of home EFP operation as the target. The target value of the QCC activity was calculated by the formula: target value = status quo value + (standard value – status quo value) x priorities for amelioration x circle capability. According to the formula, the target value was calculated to be 85.44%. According to the 80/20 rule, the Pareto chart was used to find out the main reasons that lead to the low accuracy of home EFP operation in discharged CRC patients. The Pareto analysis finally determined the root causes of the low accuracy of home EFP operation in the discharged CRC patients as training problems, pump with different types, no examination rules and lack of management, which accounted for 79.8% of the total errors (Figure 2).

The QCC head and members assessed the various measures, and also evaluated and supplemented each strategy according to a 5-point system and the principle of “5W1H” (Who, What, When, Where, Why, and How), respectively (16). According to the root causes, the positive strategies were optimized as follows.

*Improve the diversity of training and enhance the learning effect of patients and their families.* Multiple teaching styles and ways of health education were adopted based on unifying the documents and videos for discharge education on enteral nutrition. Especially, QR codes were created for easy reading and viewing by patients at any time, such as Illustrated brochures and training videos of nutrient pump operation, which could be scanned by some popular software including WeChat and Alipay.

*Uniform the type of nutrition pump.* Carry out multidisciplinary communication, cooperate with the nursing department and the medical engineering department, and unify the type of enteral nutrition pumps in the Zhejiang cancer hospital. The Department of Medical Engineering recycles old equipment and distributes unified nutrition pumps. The QCC members formulated corresponding specifications for the usage of the unified home EFP and made easy-to-understand videos for EFP operation. The nurses take action and make ensure that the patients are hospitalized with the same type and model of nutrition pump as the one at home. The patients are monitored to use a unified nutrient pump from the first day of tachymetric tube feeding until they are discharged from the hospital.

*Set up the examination rules of home EFP operation and strengthen the management of nutrition pump operation:* First, make a clear assessment plan and examination rules for home EFP operation. Secondly, set up a quality control group, establish a one-to-one accountability system, and clarify the responsibilities of each member in the group. Thirdly, the department head and the head nurse conduct regular inspections for the implementation of the one-to-one accountability system by group members.

#### Nutritional and functional status analysis

In this study, PG-SGA was used to assess and compare the nutritional status of cancer patients (28–30). Barthel Index was used to assess and compare functional status as measured (31). We used body composition analyzer (InBody770, Biospace Co., Ltd., Korea), which had been purchased by Zhejiang Cancer Hospital from the manufacturer, to measure BFM and SLM by following the company protocol.

#### Statistical processing

The sample size in the QCC program was determined by PASS 15.0 statistical software (NCSS Statistical Software, Kaysville, UT) with an 80% power setting to detect the QCC effect. The data were analyzed by SPSS 26.0 software (SPSS Inc. Chicago, IL, U.S.) after double-checking for accuracy. Measurement data that are normally distributed were expressed as mean ± standard deviation (X ± S) and counting data as percentages (%). If the measurement data conform to the normal distribution, one-way ANOVA was used to compare between groups, repeated measurement ANOVA to compare intra-group changes over time, and paired t-test were used for intra-group comparison. If the measurement data conform to the non-normal distribution, nonparametric Wilcoxon tests were used for intra-group comparison. Qualitative data were compared with the *χ*^2^-test between the two groups before and after the QCC application. *p* < 0.05 was regarded as statistically significant.

## Results

**Figure 3:**
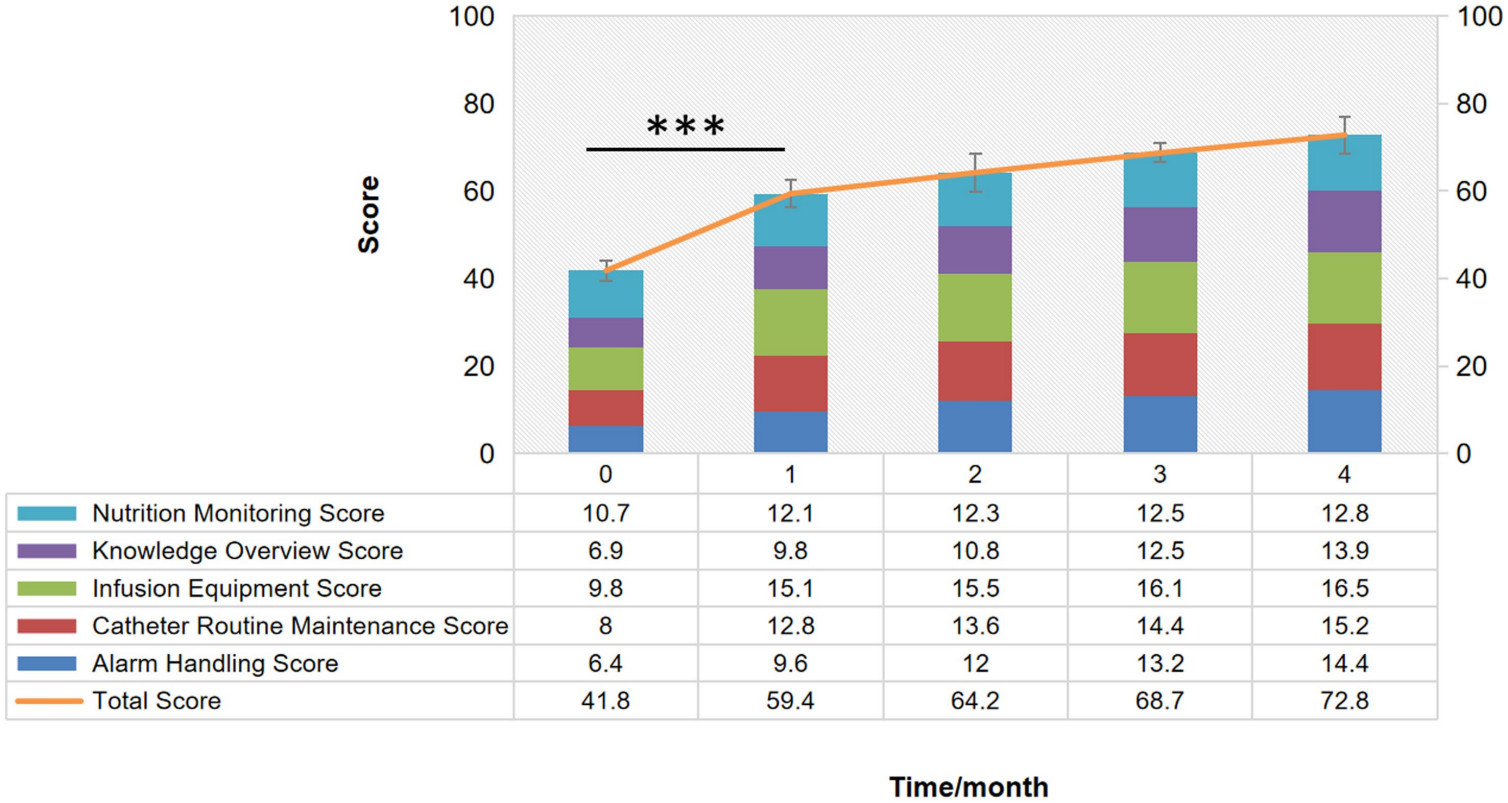
Stacked Bar of the comparison of the scores of “the nutrition pump operation checklist” before and after the QCC activity. ***p < 0.001.

To check the QCC effect on the home EFP operation, we first examined the operational performance by the comparison of the scores of the nutrition pump operation checklist before and after the QCC activity. The score was significantly increased every month since the 4-month implementation of QCC activity, including the total score and score of each item of nutrition monitoring, knowledge overview, infusion equipment, catheter routine maintenance and alarming handling (Figure 3). After the application of QCC activity, the net score increased from 41.8 to 72.8 and the fold change is 1.7. The biggest improvement appeared after the first month of QCC implementation, the total score increased from 41.8 to 59.4 with the fold change being 1.42 by both intra-group comparisons (*p* < 0.001). It showed that the operational performance of home EFP was significantly improved in discharged CRC patients by QCC activity by both intra- and inter-group comparisons.

**Figure 4:**
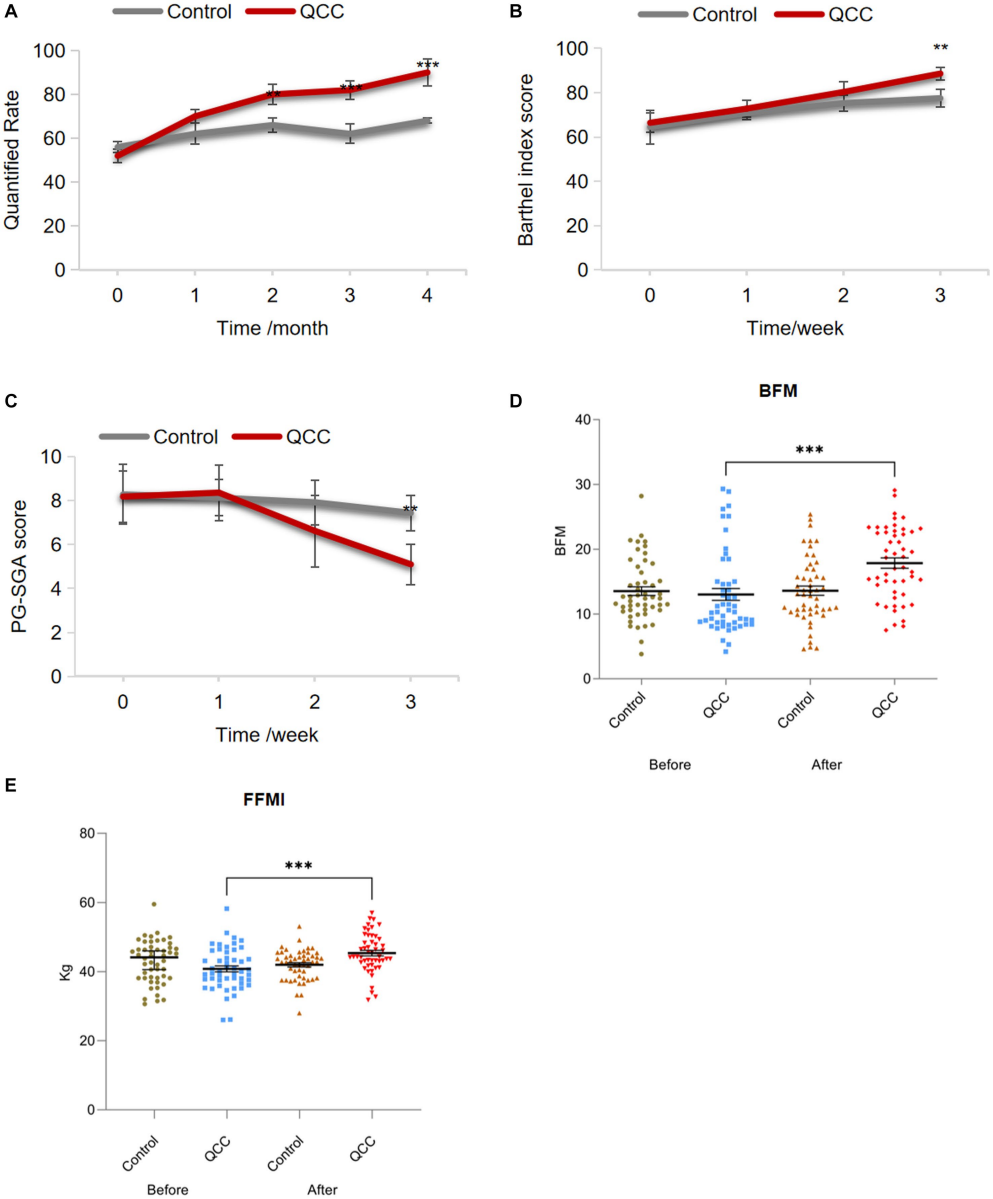
The effect of QCC activity on the pass rate of home EFP operation, the nutritional status and quality of life in discharged CRC patients. **(A)** The pass rate of home EFP operation, **(B)** BI (Barthel Index) score, **(C)** PG-SGA (Scored Patient-Generated Subjective Global Assessment) score, **(D)** BFM (Body Fat Mass), and **(E)** FFMI (Fat-Free Mass Index) upon the implementation of the QCC program. *p < 0.05, **p < 0.01, ***p < 0.001.

Next, the pass rate of home EFP operation was examined every month during the 4-month QCC activity. The pass rate significantly increased from 52 to 70% after 2 months of QCC activity (*p* < 0.01) while there was no significant difference in the control group (Figure 4A and Table 2). The pass rate gradually increased every month upon QCC implementation and even reached 90% after 4-month QCC activity. Both intra- and inter-group comparisons demonstrated the significant change induced by QCC activity, indicating the obvious improvement of operational performance of discharged CRC patients on home EFP.

**Table 2:** Comparison of pass rate of ENP performance between control and observation groups before and after QCC activity.

Pass rate	Before QCC	After QCC (month)			
		1	2	3	4
Observation group n (%)	26 (52%)	35 (70%)	40 (80%)	41 (82%)	45 (90%)
Control group n (%)	28 (56%)	31 (62%)	33 (66%)	31 (62%)	34 (68%)
*X*^2^_a_		3.405	8.734	10.176	17.533
*P*_a_		0.065	0.003	0.001	0.000
*X* ^2^_b_		0.372	1.051	0.372	1.528
*P*_b_		0.542	0.305	0.542	0.216
*X* ^2^_c_	0.161	0.7131	2.486	7.603	7.294
*P*_c_	0.688	0.398	0.115	0.006	0.007

The *X*^2^_a_ and P_a_ value indicate inter-group comparisons before and after the implementation of QCC activity in the experimental group, while the *X*^2^_b_ and P_b_ values indicate inter-group comparisons in the control group. The *X*^2^_c_ and P_c_ values indicate intra-group comparisons between the experimental group and the control group before and after QCC activity.

We then investigated the impact of QCC activity on nutritional status and quality of life in discharged CRC patients. Barthel Index score was gradually increased every month as the QCC activity was executed, and demonstrated a significant change (from 72.8 to 88.6) after 3-month QCC activity by the inter-group comparisons in both the control and experimental group, while more obvious improvements were observed in the experimental group compared to that in the control group since 2 months’ QCC activity (Figure 4B and Table 3, *p* < 0.01). It claimed that QCC activity positively affected the performance in activities of daily living (ADL) of discharged CRC patients. PG-SGA was used to evaluate the nutritional status of patients in the QCC program through interdisciplinary assessment (weight, nutrition intake, symptoms, functional status, disease state, metabolic stress and nutritional physical examination). It showed that the PG-SGA score was significantly decreased after 3 months of QCC implementation by both intra- and inter-group comparisons (Figure 4C and Table 4, *p* < 0.01), indicating the improvement of nutritional status of discharged CRC patients by QC activity. The same change was also observed in BFM (Body Fat Mass) and FFMI (Fat-Free Mass Index), which were significantly increased after QCC activity compared to that before QCC application (Figures 4D,E and Tables 5-6, *p* < 0.001). Thus, QCC activities can not only significantly increase the pass rate of EFP use and promote the effective completion of enteral nutrition support, but also improve the nutritional status and quality of life in cancer patients.

**Table 3:** Comparison of BI scores before and after the implementation of the QCC of the two groups (x ± s).

Group	Before QCC	After QCC (month)				
		1	2	3	*F* value	*P* value
Control (*n* = 50)	64.5 ± 13.60	70.8 ± 9.81	75.3 ± 8.64	77.5 ± 8.88	67.755	<0.01
Experiment (*n* = 50)	66.4 ± 13.44	72.8 ± 9.85	80.3 ± 9.55	88.6 ± 7.89	14.083	<0.01
*t* value	−0.703	−1.017	−2.743	−6.607	–	–
*P* value	0.484	0.312	0.007	0.000	–	–

**Table 4:** Comparison of PG-SGA scores before and after the implementation of the QCC of the two groups (x ± s).

Group	Before QCC	After QCC (month)				
		1	2	3	*F* value	*P* value
Control (*n* = 50)	8.08 ± 2.18	8.08 ± 2.27	7.92 ± 2.63	7.44 ± 2.40	13.517	<0.01
Experiment (*n* = 50)	8.18 ± 2.36	8.36 ± 2.83	6.62 ± 2.02	5.40 ± 2.36	7.681	0.007
*t* value	−0.207	−0.504	0.448	2.868	–	–
*P* value	0.836	0.590	0.627	0.005	–	–

**Table 5:** Comparison of body fat mass (BFM) before and after the implementation of 3-month quality control circles in the two groups (Mean ± SD, kg).

Group	Before QCC	After QCC	*t* value	*P* value
Control (*n* = 50)	13.534 ± 4.70	13.604 ± 5.06	0.071	0.9436
Experiment (*n* = 50)	13.012 ± 6.39	17.886 ± 5.51	4.026	0.0001
*t* value	0.461	3.986	–	–
*P* value	0.6462	0.0001	–	–

**Table 6:** Comparison of superior longitudinal muscle (SLM) before and after the implementation of 3-month quality control circles in the two groups (Mean ± SD, kg).

Group	Before QCC	After QCC	*t* value	*P* value
Control (*n* = 50)	42.816 ± 6.09	41.962 ± 4.43	0.7937	0.4293
Experiment (*n* = 50)	40.786 ± 6.05	45.376 ± 5.63	3.891	0.0002
*t* value	1.655	3.338	–	–
*P* value	0.1010	0.0012	–	–

## Discussion

Malnutrition directly affects the prognosis, case fatality and incidence of complications of cancer patients (32). To reduce the economic burden of patients, improve their quality of life and increase time spent with their families, home nutrition support was provided under the guidance of doctors and nurses. The use of enteral nutrition is more prevalent compared to parenteral nutrition due to the higher technical requirements and the susceptibility to complications in parenteral nutrition. Enteral nutrition can benefit the structure and function of the gastrointestinal mucosa, maintain intestinal integrity, promote intestinal peristalsis, meet the physiological needs of patients, block the vicious circle of malnutrition and immunodeficiency, and be more conducive to the recovery of patients (33). Improper use of enteral nutrition may cause complications such as diarrhea, gastric retention, and aspiration, while home EFP could decrease the incidence of these complications. At present, due to the imperfect intervention of doctors and nurses for patients who have been discharged, patients and their families are not familiar with the operation of enteral nutrition pumps. It results in the low accuracy rate of enteral nutrition pump operation at home, which in turn affects the utilization rate of home EFP by patients (34). To improve the accuracy rate of home EFP operation, we established a quality control circle with a medical and nursing combination, aimed to scientifically analyze the reasons for the low pass rate of home EFP operation, and implement targeted countermeasures to improve the qualification rate of nutrition pump.

In this study, the data showed that QC activity improved the accuracy of home EFP operation. The pass rate of home EFP operation was only 52% before QCC activity, and it increased to 70, 80, 82 and 90% after 1, 2, 3 and 4 months of implementation of the countermeasures, respectively. The target completion rate exceeded the set value of 85.44% and increased month by month at the 4-month QCC implementation stage. Basically, from the third month, there was a significant difference in the pass rate of the home EFP operation. It demonstrated that QCC activities carried out by both doctors and nurses play a positive role in some hospital services, consistent with the previous studies (35, 36, 38), should be applied to solve more clinical problems and benefit more cancer patients.

In this activity, team members understood the status quo and investigated the potential reasons for the low accuracy by data review and brainstorm. The root causes of low accuracy included training problems, inconsistent types of pumps, lack of examination rules and lack of management. After implementing the targeted measurements, the pass rate of home EFP operation was significantly increased. The success in the improvement of home EFP operation then subsequently improved the nutritional status and quality of life of cancer patients, which were validated by the positive changes in the indicators such as BI score, PG-SGA score, BFM and FFMI. Interestingly, we found that the changes of the indicators slowdown in the later period of QCC activity. Patients and their families may be tired of studying because of the exhaustion of energy and interest as time passed in the long-time QCC activity. Therefore, for patients who have been using home enteral nutrition pumps for a long time, the supervision and intervention measurements of the quality control circle cannot be static and should be adjusted for different stages.

In addition, during the development of this study, we found that the QCC program strengthened the hospital-patient interaction and effectively improved patient satisfaction. Since the QCC activity emphasized the automatic and spontaneous participation of members in the circle, QCC members enjoyed higher autonomy, participation and management, and passion at work has also been enhanced. Also, the ability to innovate, scientific research thinking and planning ability has been improved in the process of participation.

This study was conducted by the collaboration of multiple departments in a large specialized oncology hospital. However, it also has some limitations. First of all, considering the economy and feasibility, the patients in this study were all from the same hospital and had a limited sample size, leading to a lack of generality. A multi-center study with a big sample size will bring more scientific and unbiased results, which could be carried out in the future. Also, studies were conducted for only 7 months and can not reflect longer-term effects. The improvement effect on accuracy and nutritional status was flattened at the end of QCC activity. A long-term study may help to find out more potential problems in the study design and implementation.

## Data availability statement

The raw data supporting the conclusions of this article will be made available by the authors, without undue reservation.

## Ethics statement

The studies involving human participants were reviewed and approved by the Research Ethic Committee of Zhejiang Cancer University. The patients/participants provided their written informed consent to participate in this study.

## Author contributions

YoL, LL, and YiL conceived the study and drafted the paper. XY, XN, WS, QZ, and FC coordinated and performed the data collection. YoL and XY contributed to the conception and design of the study. LL and YiL provided critical revision of the manuscript. All authors contributed to the article and approved the submitted version.
