# Supplementary figures and images for: A quality control circle process to improve enteral nutrition feeding support in discharged patients with colorectal cancer
Source: Front Nutr. 2023 Jul 19;10:1191804. doi: 10.3389/fnut.2023.1191804 (PMC10396396; doi:10.3389/fnut.2023.1191804)

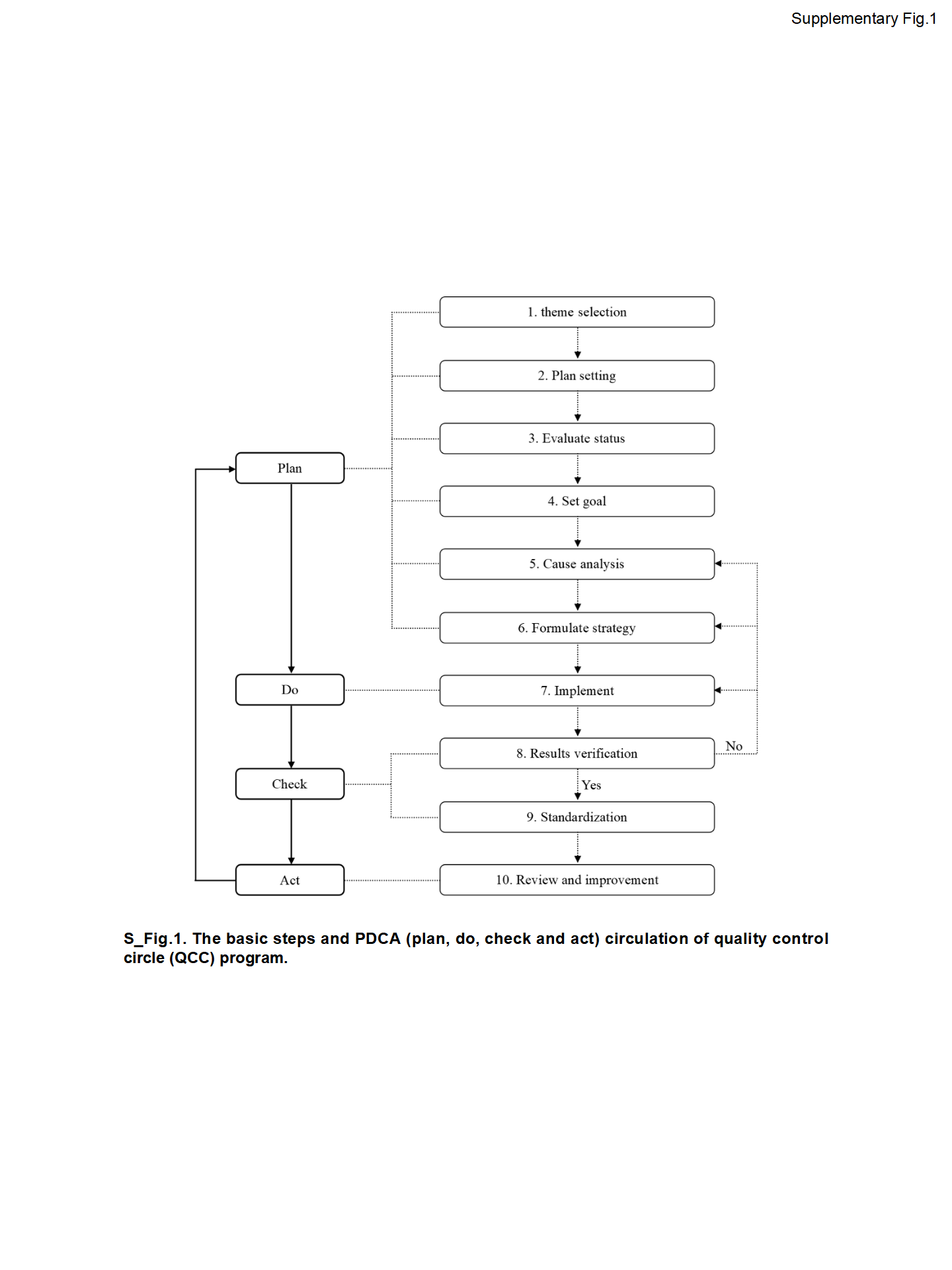

Supplement: Supplementary file 1 [file Image_1.TIF]
